# Bis(η^5^-1-*tert*-butyl­inden­yl)nickel(II)

**DOI:** 10.1107/S1600536811028510

**Published:** 2011-08-02

**Authors:** Heiko Bauer, Yu Sun, Helmut Sitzmann

**Affiliations:** a Department of Chemistry, Technical University of Kaiserslautern, 67663 Kaiserslautern, Germany

## Abstract

The title compound, [Ni(C_13_H_15_)_2_], shows a slightly distorted sandwich structure with two independent mol­ecules in the asymmetric unit. Both Ni atoms are located on crystallographic centres of inversion.

## Related literature

For the synthetic procedure of the analogous indenylcobalt complex, see: Gou *et al.* (2007 ▶). For a description of the Cambridge Structural Database, see: Allen (2002 ▶). For the use of bis­(inden­yl)nickel(II) complexes as starting compounds for poly- and oligomerization catalysts, see: Xie *et al.* (2009 ▶); Fontaine & Zargarian *et al.* (2004 ▶). For the indenyl effect in S_N_1, S_N_2 and other reactions, see: Elschenbroich (2008 ▶); Rerek & Basolo (1984 ▶); Rerek *et al.* (1983 ▶), O’Connor & Casey (1987 ▶); Turaki *et al.* (1988 ▶); Caddy *et al.* (1978 ▶); Bönnemann (1985 ▶); Marder *et al.* (1988 ▶).
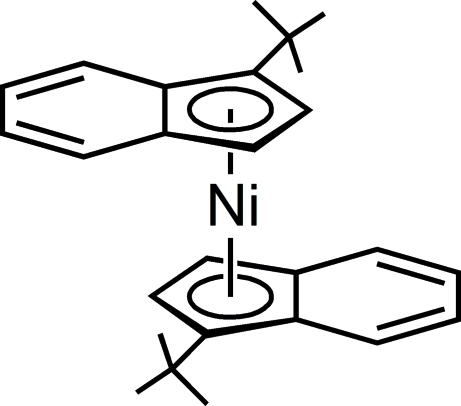


## Experimental

### 

#### Crystal data

[Ni(C_13_H_15_)_2_]
*M* *_r_* = 401.21
Triclinic, 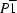

*a* = 9.8116 (5) Å
*b* = 10.9631 (7) Å
*c* = 11.1658 (7) Å
α = 68.800 (6)°
β = 67.085 (5)°
γ = 85.212 (4)°
*V* = 1029.10 (11) Å^3^
*Z* = 2
Cu *K*α radiation
μ = 1.38 mm^−1^
*T* = 150 K
0.11 × 0.07 × 0.04 mm

#### Data collection

Oxford Diffraction Xcalibur Sapphire3 Gemini ultra diffractometer
Absorption correction: multi-scan (*CrysAlis PRO*; Oxford Diffraction, 2010 ▶) *T*_min_ = 0.675, *T*_max_ = 1.000
8813 measured reflections
3283 independent reflections
2838 reflections with *I* > 2σ(*I*)
*R*_int_ = 0.022

#### Refinement

*R*[*F* ^2^ > 2σ(*F* ^2^)] = 0.027
*wR*(*F* ^2^) = 0.075
*S* = 1.07
3283 reflections
253 parameters
H-atom parameters constrained
Δρ_max_ = 0.24 e Å^−3^
Δρ_min_ = −0.25 e Å^−3^

### 

Data collection: *CrysAlis CCD* (Oxford Diffraction, 2010 ▶); cell refinement: *CrysAlis RED* (Oxford Diffraction, 2010 ▶); data reduction: *CrysAlis RED*; program(s) used to solve structure: *SIR92* (Altomare *et al.*, 1994 ▶); program(s) used to refine structure: *SHELXL97* (Sheldrick, 2008 ▶); molecular graphics: *SCHAKAL99* (Keller, 1999 ▶); software used to prepare material for publication: *publCIF* (Westrip, 2010 ▶).

## Supplementary Material

Crystal structure: contains datablock(s) I, global. DOI: 10.1107/S1600536811028510/im2294sup1.cif

Structure factors: contains datablock(s) I. DOI: 10.1107/S1600536811028510/im2294Isup2.hkl

Additional supplementary materials:  crystallographic information; 3D view; checkCIF report

## Figures and Tables

**Fig. 1.**
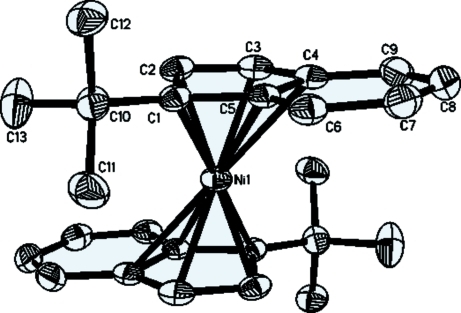
View of the title compound showing thermal ellipsoids at the 50% probability level.

**Fig. 2.**
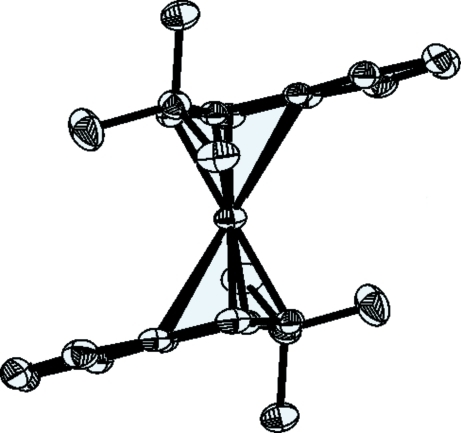
View of the title compound showing the folding angle of the indenyl ligand.

## supplementary crystallographic information

### Comment

The catalytic activity of indenylnickel(II) complexes has stimulated recent research activity. It is e.g. possible to oligomerize phenylsilane in the presence of a methylindenylnickel(II) phosphine complex (Fontaine & Zargarian, 2004). *N*-heterocyclic carbene complexes of indenylnickel(II) chloride have been shown to polymerize styrene (Xie *et al.*, 2009). The starting compounds for these complexes are bis(indenyl)nickel(II) and bis(1-methylindenyl)nickel(II). With the well known indenyl effect (Elschenbroich, 2008) based on a slip-fold distortion of the indenyl ligand from *η*^5^ to *η*^3^ coordination, which greatly enhances the reactivity in S_N_1 and S_N_2 substitution reactions (Rerek & Basolo 1984, Rerek *et al.* 1983; O'Connor & Casey, 1987; Turaki *et al.*, 1988) and other reactions, (Caddy *et al.*, 1978; Bönnemann, 1985; Marder *et al.*, 1988) the as yet unknown bis(*η*^5^-1-*tert*-butylindenyl)nickel(II) complex became interesting to us as a promising starting compound.

The title compound was synthesized from lithium 1-*tert*-butylindenide and nickel(II) bromide dimethoxyethane complex and crystallized as dark red prisms. In the structure shown here, the nickel atom is bound to the carbon atoms of the five-membered ring of the ligand by a distorted *η*^5^-coordination. The metal ion is positioned on a crystallographic centre of inversion and the two indenyl ligands are therefore arranged in a staggered coordination with a rotation angle of 180°. As known from similar complexes (Gou *et al.*, 2007), the lengths of the five Ni—C bonds are split in two sets. The three shorter bond distances are 2.124 (1) Å, 1.994 (2) Å and 2.049 (2) Å, the two longer bond distances are 2.460 (2) Å and 2.505 (1) Å, which is even longer than for the Co complex (Gou *et al.*, 2007). The distance between the nickel centre and the centroid of the five-membered ring is 1.7950 (8) Å. The folding angle of 4.28 (7)° shows a slightly smaller value than for unsubstituted indenyl complexes (Rerek *et al.*, 1983). The two different bond length ranges are in accordance with the usual *η*^2^+*η*^3^-coordination of the indenyl ligand resulting from the reluctant participation of the benzene ring in the ligand-metal electron donation (Rerek *et al.*, 1983). The bond between C1 and C10 is bending out 7.2 (1)° of the ring plane.

### Experimental

To a stirred solution of 1-*tert*-butylindene (862 mg, 5.0 mmol) in diethyl ether (10 ml) a solution of n-BuLi (1.6 mol/l, 3.44 ml, 5.5 mmol) in hexane was added slowly at 0 °C. Stirring was continued for 19 h at room temperature, then the solvent was removed *in vacuo*. The resulting white precipitate was suspended in pentane, cooled overnight in a fridge, filtered and washed with pentane. The lithium 1-*tert*-butylindenide was suspended in THF (10 ml) and NiBr_2_ × dme (1.54 g, 5.0 mmol) was added. The mixture was stirred for 24 h at room temperature. The solvent was removed *in vacuo* and the residue extracted with pentane. The product was obtained as dark red prisms at -30 °C (323 mg, 16%).

### Refinement

All hydrogen atoms were placed in calculated positions (C—H 0.95 or 0.98 Å) and refined by using a riding model, with *U*_iso_(H)=1.2–1.5 *U*_eq_ of the parent atom.

### Crystal data

**Table:**

[Ni(C_13_H_15_)_2_]	*Z* = 2
*M**_r_* = 401.21	*F*(000) = 428
Triclinic, *P*1	*D*_x_ = 1.295 Mg m^−^^3^
Hall symbol: -P 1	Cu *K*α radiation, λ = 1.54184 Å
*a* = 9.8116 (5) Å	Cell parameters from 5554 reflections
*b* = 10.9631 (7) Å	θ = 4.3–62.6°
*c* = 11.1658 (7) Å	µ = 1.38 mm^−^^1^
α = 68.800 (6)°	*T* = 150 K
β = 67.085 (5)°	Transparent prism, red
γ = 85.212 (4)°	0.11 × 0.07 × 0.04 mm
*V* = 1029.10 (11) Å^3^	

### Data collection

**Table:**

Oxford Diffraction Xcalibur Sapphire3 Gemini ultra diffractometer	3283 independent reflections
Radiation source: Enhance Ultra (Cu) X-ray Source	2838 reflections with *I* > 2σ(*I*)
mirror	*R*_int_ = 0.022
Detector resolution: 16.1399 pixels mm^-1^	θ_max_ = 62.6°, θ_min_ = 4.3°
ω scans	*h* = −9→11
Absorption correction: multi-scan (*CrysAlis PRO*; Oxford Diffraction, 2010)	*k* = −12→12
*T*_min_ = 0.675, *T*_max_ = 1.000	*l* = −12→12
8813 measured reflections	

### Refinement

**Table:**

Refinement on *F*^2^	Primary atom site location: structure-invariant direct methods
Least-squares matrix: full	Secondary atom site location: difference Fourier map
*R*[*F*^2^ > 2σ(*F*^2^)] = 0.027	Hydrogen site location: inferred from neighbouring sites
*wR*(*F*^2^) = 0.075	H-atom parameters constrained
*S* = 1.07	*w* = 1/[σ^2^(*F*_o_^2^) + (0.049*P*)^2^] where *P* = (*F*_o_^2^ + 2*F*_c_^2^)/3
3283 reflections	(Δ/σ)_max_ < 0.001
253 parameters	Δρ_max_ = 0.24 e Å^−^^3^
0 restraints	Δρ_min_ = −0.25 e Å^−^^3^

### Special details

**Table:**

Experimental. CrysAlisPro, Oxford Diffraction Ltd., Version 1.171.33.66 (release 28-04-2010 CrysAlis171 .NET) (compiled Apr 28 2010,14:27:37) Empirical absorption correction using spherical harmonics, implemented in SCALE3 ABSPACK scaling algorithm.
Geometry. All s.u.'s (except the s.u. in the dihedral angle between two l.s. planes) are estimated using the full covariance matrix. The cell s.u.'s are taken into account individually in the estimation of s.u.'s in distances, angles and torsion angles; correlations between s.u.'s in cell parameters are only used when they are defined by crystal symmetry. An approximate (isotropic) treatment of cell s.u.'s is used for estimating s.u.'s involving l.s. planes.
Refinement. Refinement of *F*^2^ against ALL reflections. The weighted *R*-factor *wR* and goodness of fit *S* are based on *F*^2^, conventional *R*-factors *R* are based on *F*, with *F* set to zero for negative *F*^2^. The threshold expression of *F*^2^ > 2σ(*F*^2^) is used only for calculating *R*-factors(gt) *etc*. and is not relevant to the choice of reflections for refinement. *R*-factors based on *F*^2^ are statistically about twice as large as those based on *F*, and *R*- factors based on ALL data will be even larger.

### Fractional atomic coordinates and isotropic or equivalent isotropic displacement parameters (Å^2^)

**Table:**

	*x*	*y*	*z*	*U*_iso_*/*U*_eq_	
C1	0.69298 (15)	0.98880 (15)	0.83091 (15)	0.0224 (3)	
C2	0.66605 (16)	1.11686 (15)	0.83603 (16)	0.0253 (3)	
H2	0.7279	1.1686	0.8495	0.030*	
C3	0.53173 (17)	1.15413 (15)	0.81780 (16)	0.0262 (3)	
H3	0.4779	1.2263	0.8348	0.031*	
C4	0.49048 (16)	1.06274 (15)	0.76852 (15)	0.0246 (3)	
C5	0.59070 (15)	0.96046 (14)	0.77499 (15)	0.0215 (3)	
C6	0.57746 (17)	0.85971 (15)	0.73069 (16)	0.0265 (3)	
H6	0.6427	0.7906	0.7348	0.032*	
C7	0.46801 (18)	0.86187 (17)	0.68070 (17)	0.0334 (4)	
H7	0.4605	0.7948	0.6481	0.040*	
C8	0.36868 (18)	0.96052 (18)	0.67725 (18)	0.0360 (4)	
H8	0.2937	0.9589	0.6438	0.043*	
C9	0.37800 (17)	1.06062 (17)	0.72184 (17)	0.0325 (4)	
H9	0.3091	1.1269	0.7208	0.039*	
C10	0.83254 (16)	0.91731 (16)	0.83483 (17)	0.0273 (3)	
C11	0.79792 (18)	0.76942 (17)	0.91908 (18)	0.0370 (4)	
H11A	0.7370	0.7558	1.0169	0.056*	
H11B	0.8908	0.7264	0.9126	0.056*	
H11C	0.7439	0.7319	0.8815	0.056*	
C12	0.93683 (17)	0.93709 (17)	0.68371 (17)	0.0325 (4)	
H12A	0.8883	0.8985	0.6426	0.049*	
H12B	1.0288	0.8943	0.6829	0.049*	
H12C	0.9598	1.0311	0.6294	0.049*	
C13	0.91121 (19)	0.9744 (2)	0.8992 (2)	0.0457 (5)	
H13A	0.9415	1.0670	0.8421	0.069*	
H13B	0.9990	0.9260	0.9029	0.069*	
H13C	0.8435	0.9666	0.9937	0.069*	
C14	1.06039 (15)	0.56135 (14)	0.63293 (15)	0.0207 (3)	
C15	1.03662 (17)	0.66575 (14)	0.52347 (16)	0.0246 (3)	
H15	1.1096	0.7312	0.4502	0.030*	
C16	0.88655 (18)	0.65609 (16)	0.54168 (19)	0.0304 (4)	
H16	0.8453	0.7033	0.4752	0.036*	
C17	0.80661 (17)	0.56158 (16)	0.67958 (19)	0.0305 (4)	
C18	0.91244 (16)	0.50176 (15)	0.73735 (16)	0.0247 (3)	
C19	0.86545 (19)	0.40421 (17)	0.86990 (17)	0.0354 (4)	
H19	0.9352	0.3626	0.9090	0.042*	
C20	0.7145 (2)	0.3692 (2)	0.9435 (2)	0.0499 (6)	
H20	0.6813	0.3043	1.0344	0.060*	
C21	0.6116 (2)	0.4273 (2)	0.8868 (3)	0.0561 (7)	
H21	0.5092	0.4011	0.9395	0.067*	
C22	0.65523 (18)	0.5224 (2)	0.7551 (2)	0.0463 (5)	
H22	0.5842	0.5607	0.7165	0.056*	
C23	1.20379 (16)	0.54344 (15)	0.65758 (16)	0.0236 (3)	
C24	1.18369 (17)	0.58486 (17)	0.78106 (17)	0.0300 (4)	
H24A	1.1568	0.6761	0.7607	0.045*	
H24B	1.2767	0.5767	0.7959	0.045*	
H24C	1.1048	0.5281	0.8652	0.045*	
C25	1.24681 (18)	0.40112 (16)	0.69020 (18)	0.0338 (4)	
H25A	1.1628	0.3430	0.7666	0.051*	
H25B	1.3318	0.3913	0.7176	0.051*	
H25C	1.2734	0.3780	0.6073	0.051*	
C26	1.33151 (17)	0.63006 (17)	0.52843 (18)	0.0339 (4)	
H26A	1.3410	0.6090	0.4475	0.051*	
H26B	1.4241	0.6140	0.5439	0.051*	
H26C	1.3110	0.7225	0.5114	0.051*	
Ni1	0.5000	1.0000	1.0000	0.02238 (12)	
Ni2	1.0000	0.5000	0.5000	0.02285 (12)	

### Atomic displacement parameters (Å^2^)

**Table:**

	*U*^11^	*U*^22^	*U*^33^	*U*^12^	*U*^13^	*U*^23^
C1	0.0222 (7)	0.0248 (8)	0.0166 (8)	−0.0013 (6)	−0.0025 (6)	−0.0083 (6)
C2	0.0280 (8)	0.0250 (8)	0.0182 (8)	−0.0055 (6)	−0.0017 (6)	−0.0091 (6)
C3	0.0313 (8)	0.0201 (8)	0.0194 (8)	0.0025 (6)	−0.0032 (6)	−0.0057 (6)
C4	0.0264 (8)	0.0242 (8)	0.0156 (8)	0.0000 (6)	−0.0033 (6)	−0.0034 (6)
C5	0.0221 (7)	0.0226 (8)	0.0139 (7)	−0.0017 (6)	−0.0020 (6)	−0.0049 (6)
C6	0.0305 (8)	0.0248 (8)	0.0207 (8)	−0.0012 (6)	−0.0053 (6)	−0.0085 (6)
C7	0.0383 (9)	0.0367 (10)	0.0245 (9)	−0.0108 (7)	−0.0084 (7)	−0.0113 (7)
C8	0.0307 (9)	0.0481 (11)	0.0264 (9)	−0.0070 (8)	−0.0129 (7)	−0.0063 (8)
C9	0.0287 (8)	0.0371 (10)	0.0248 (9)	0.0035 (7)	−0.0100 (7)	−0.0044 (7)
C10	0.0227 (7)	0.0359 (9)	0.0235 (8)	0.0047 (6)	−0.0064 (6)	−0.0143 (7)
C11	0.0317 (8)	0.0390 (10)	0.0293 (9)	0.0106 (7)	−0.0085 (7)	−0.0053 (8)
C12	0.0258 (8)	0.0367 (10)	0.0273 (9)	0.0042 (7)	−0.0020 (7)	−0.0125 (7)
C13	0.0297 (9)	0.0751 (14)	0.0486 (12)	0.0102 (9)	−0.0185 (8)	−0.0381 (11)
C14	0.0223 (7)	0.0202 (7)	0.0206 (8)	0.0012 (6)	−0.0063 (6)	−0.0105 (6)
C15	0.0310 (8)	0.0179 (7)	0.0281 (9)	0.0035 (6)	−0.0135 (7)	−0.0100 (6)
C16	0.0349 (9)	0.0266 (8)	0.0450 (10)	0.0138 (7)	−0.0253 (8)	−0.0218 (8)
C17	0.0241 (8)	0.0349 (9)	0.0464 (11)	0.0071 (7)	−0.0121 (7)	−0.0326 (8)
C18	0.0237 (7)	0.0270 (8)	0.0253 (8)	−0.0008 (6)	−0.0032 (6)	−0.0176 (7)
C19	0.0413 (9)	0.0367 (9)	0.0245 (9)	−0.0125 (7)	−0.0011 (7)	−0.0163 (7)
C20	0.0506 (12)	0.0551 (12)	0.0342 (11)	−0.0287 (10)	0.0123 (9)	−0.0295 (10)
C21	0.0280 (9)	0.0732 (15)	0.0689 (16)	−0.0203 (10)	0.0151 (10)	−0.0591 (14)
C22	0.0226 (8)	0.0568 (12)	0.0778 (16)	0.0058 (8)	−0.0102 (9)	−0.0551 (13)
C23	0.0224 (7)	0.0282 (8)	0.0220 (8)	0.0026 (6)	−0.0090 (6)	−0.0106 (7)
C24	0.0292 (8)	0.0371 (9)	0.0284 (9)	0.0007 (7)	−0.0121 (7)	−0.0155 (7)
C25	0.0375 (9)	0.0353 (9)	0.0373 (10)	0.0131 (7)	−0.0233 (8)	−0.0152 (8)
C26	0.0237 (8)	0.0468 (11)	0.0299 (9)	−0.0033 (7)	−0.0077 (7)	−0.0137 (8)
Ni1	0.02328 (19)	0.0207 (2)	0.0179 (2)	0.00272 (14)	−0.00330 (15)	−0.00650 (15)
Ni2	0.0256 (2)	0.0215 (2)	0.0289 (2)	0.00832 (14)	−0.01484 (16)	−0.01392 (16)

### Geometric parameters (Å, °)

**Table:**

C1—C2	1.424 (2)	C15—Ni2	2.0066 (15)
C1—C5	1.474 (2)	C15—H15	0.9500
C1—C10	1.528 (2)	C16—C17	1.449 (2)
C1—Ni1	2.1237 (14)	C16—Ni2	2.0588 (15)
C2—C3	1.416 (2)	C16—H16	0.9500
C2—Ni1	1.9942 (15)	C17—C22	1.404 (2)
C2—H2	0.9500	C17—C18	1.424 (2)
C3—C4	1.453 (2)	C17—Ni2	2.4247 (15)
C3—Ni1	2.0492 (15)	C18—C19	1.397 (2)
C3—H3	0.9500	C18—Ni2	2.4543 (15)
C4—C9	1.397 (2)	C19—C20	1.391 (3)
C4—C5	1.429 (2)	C19—H19	0.9500
C4—Ni1	2.4604 (15)	C20—C21	1.387 (3)
C5—C6	1.397 (2)	C20—H20	0.9500
C5—Ni1	2.5048 (14)	C21—C22	1.382 (3)
C6—C7	1.385 (2)	C21—H21	0.9500
C6—H6	0.9500	C22—H22	0.9500
C7—C8	1.394 (3)	C23—C25	1.531 (2)
C7—H7	0.9500	C23—C26	1.534 (2)
C8—C9	1.381 (3)	C23—C24	1.540 (2)
C8—H8	0.9500	C24—H24A	0.9800
C9—H9	0.9500	C24—H24B	0.9800
C10—C13	1.530 (2)	C24—H24C	0.9800
C10—C11	1.539 (2)	C25—H25A	0.9800
C10—C12	1.539 (2)	C25—H25B	0.9800
C11—H11A	0.9800	C25—H25C	0.9800
C11—H11B	0.9800	C26—H26A	0.9800
C11—H11C	0.9800	C26—H26B	0.9800
C12—H12A	0.9800	C26—H26C	0.9800
C12—H12B	0.9800	Ni1—C2^i^	1.9942 (15)
C12—H12C	0.9800	Ni1—C3^i^	2.0492 (15)
C13—H13A	0.9800	Ni1—C1^i^	2.1237 (14)
C13—H13B	0.9800	Ni1—C4^i^	2.4604 (15)
C13—H13C	0.9800	Ni1—C5^i^	2.5048 (14)
C14—C15	1.423 (2)	Ni2—C15^ii^	2.0066 (15)
C14—C18	1.477 (2)	Ni2—C16^ii^	2.0588 (15)
C14—C23	1.520 (2)	Ni2—C14^ii^	2.1166 (14)
C14—Ni2	2.1166 (14)	Ni2—C17^ii^	2.4247 (15)
C15—C16	1.413 (2)	Ni2—C18^ii^	2.4543 (15)
			
C2—C1—C5	106.58 (13)	C14—C23—C24	108.71 (12)
C2—C1—C10	125.06 (14)	C25—C23—C24	109.00 (13)
C5—C1—C10	125.43 (13)	C26—C23—C24	108.56 (13)
C2—C1—Ni1	64.95 (8)	C23—C24—H24A	109.5
C5—C1—Ni1	86.25 (8)	C23—C24—H24B	109.5
C10—C1—Ni1	128.67 (11)	H24A—C24—H24B	109.5
C3—C2—C1	108.60 (13)	C23—C24—H24C	109.5
C3—C2—Ni1	71.60 (9)	H24A—C24—H24C	109.5
C1—C2—Ni1	74.74 (9)	H24B—C24—H24C	109.5
C3—C2—H2	125.7	C23—C25—H25A	109.5
C1—C2—H2	125.7	C23—C25—H25B	109.5
Ni1—C2—H2	119.7	H25A—C25—H25B	109.5
C2—C3—C4	108.12 (13)	C23—C25—H25C	109.5
C2—C3—Ni1	67.43 (9)	H25A—C25—H25C	109.5
C4—C3—Ni1	87.54 (9)	H25B—C25—H25C	109.5
C2—C3—H3	125.9	C23—C26—H26A	109.5
C4—C3—H3	125.9	C23—C26—H26B	109.5
Ni1—C3—H3	111.3	H26A—C26—H26B	109.5
C9—C4—C5	120.57 (14)	C23—C26—H26C	109.5
C9—C4—C3	132.15 (14)	H26A—C26—H26C	109.5
C5—C4—C3	107.27 (13)	H26B—C26—H26C	109.5
C9—C4—Ni1	134.13 (11)	C2—Ni1—C2^i^	180.0
C5—C4—Ni1	74.99 (8)	C2—Ni1—C3	40.96 (6)
C3—C4—Ni1	56.32 (8)	C2^i^—Ni1—C3	139.04 (6)
C6—C5—C4	119.25 (14)	C2—Ni1—C3^i^	139.04 (6)
C6—C5—C1	133.14 (14)	C2^i^—Ni1—C3^i^	40.96 (6)
C4—C5—C1	107.61 (13)	C3—Ni1—C3^i^	180.00 (8)
C6—C5—Ni1	137.16 (11)	C2—Ni1—C1^i^	139.68 (6)
C4—C5—Ni1	71.58 (8)	C2^i^—Ni1—C1^i^	40.32 (6)
C1—C5—Ni1	57.78 (7)	C3—Ni1—C1^i^	112.92 (6)
C7—C6—C5	119.14 (15)	C3^i^—Ni1—C1^i^	67.08 (6)
C7—C6—H6	120.4	C2—Ni1—C1	40.32 (6)
C5—C6—H6	120.4	C2^i^—Ni1—C1	139.68 (6)
C6—C7—C8	121.33 (16)	C3—Ni1—C1	67.08 (6)
C6—C7—H7	119.3	C3^i^—Ni1—C1	112.92 (6)
C8—C7—H7	119.3	C1^i^—Ni1—C1	180.000 (2)
C9—C8—C7	120.81 (15)	C2—Ni1—C4	61.80 (6)
C9—C8—H8	119.6	C2^i^—Ni1—C4	118.20 (6)
C7—C8—H8	119.6	C3—Ni1—C4	36.15 (6)
C8—C9—C4	118.85 (15)	C3^i^—Ni1—C4	143.85 (6)
C8—C9—H9	120.6	C1^i^—Ni1—C4	119.04 (5)
C4—C9—H9	120.6	C1—Ni1—C4	60.96 (5)
C1—C10—C13	110.58 (13)	C2—Ni1—C4^i^	118.20 (6)
C1—C10—C11	112.17 (13)	C2^i^—Ni1—C4^i^	61.80 (6)
C13—C10—C11	108.40 (15)	C3—Ni1—C4^i^	143.85 (6)
C1—C10—C12	107.69 (13)	C3^i^—Ni1—C4^i^	36.15 (6)
C13—C10—C12	108.98 (14)	C1^i^—Ni1—C4^i^	60.96 (5)
C11—C10—C12	108.97 (14)	C1—Ni1—C4^i^	119.04 (5)
C10—C11—H11A	109.5	C4—Ni1—C4^i^	180.0
C10—C11—H11B	109.5	C2—Ni1—C5^i^	119.05 (5)
H11A—C11—H11B	109.5	C2^i^—Ni1—C5^i^	60.95 (5)
C10—C11—H11C	109.5	C3—Ni1—C5^i^	119.72 (5)
H11A—C11—H11C	109.5	C3^i^—Ni1—C5^i^	60.28 (5)
H11B—C11—H11C	109.5	C1^i^—Ni1—C5^i^	35.97 (5)
C10—C12—H12A	109.5	C1—Ni1—C5^i^	144.03 (5)
C10—C12—H12B	109.5	C4—Ni1—C5^i^	146.56 (5)
H12A—C12—H12B	109.5	C4^i^—Ni1—C5^i^	33.44 (5)
C10—C12—H12C	109.5	C2—Ni1—C5	60.95 (5)
H12A—C12—H12C	109.5	C2^i^—Ni1—C5	119.05 (5)
H12B—C12—H12C	109.5	C3—Ni1—C5	60.28 (5)
C10—C13—H13A	109.5	C3^i^—Ni1—C5	119.72 (5)
C10—C13—H13B	109.5	C1^i^—Ni1—C5	144.03 (5)
H13A—C13—H13B	109.5	C1—Ni1—C5	35.97 (5)
C10—C13—H13C	109.5	C4—Ni1—C5	33.44 (5)
H13A—C13—H13C	109.5	C4^i^—Ni1—C5	146.56 (5)
H13B—C13—H13C	109.5	C5^i^—Ni1—C5	180.000 (1)
C15—C14—C18	106.70 (12)	C15—Ni2—C15^ii^	180.00 (9)
C15—C14—C23	125.15 (13)	C15—Ni2—C16	40.67 (6)
C18—C14—C23	125.70 (13)	C15^ii^—Ni2—C16	139.33 (6)
C15—C14—Ni2	65.71 (8)	C15—Ni2—C16^ii^	139.33 (6)
C18—C14—Ni2	84.13 (9)	C15^ii^—Ni2—C16^ii^	40.67 (6)
C23—C14—Ni2	128.84 (10)	C16—Ni2—C16^ii^	180.000 (1)
C16—C15—C14	108.81 (14)	C15—Ni2—C14	40.25 (6)
C16—C15—Ni2	71.65 (9)	C15^ii^—Ni2—C14	139.75 (6)
C14—C15—Ni2	74.04 (8)	C16—Ni2—C14	67.03 (6)
C16—C15—H15	125.6	C16^ii^—Ni2—C14	112.97 (6)
C14—C15—H15	125.6	C15—Ni2—C14^ii^	139.75 (6)
Ni2—C15—H15	120.4	C15^ii^—Ni2—C14^ii^	40.25 (6)
C15—C16—C17	107.97 (14)	C16—Ni2—C14^ii^	112.97 (6)
C15—C16—Ni2	67.68 (8)	C16^ii^—Ni2—C14^ii^	67.03 (6)
C17—C16—Ni2	85.57 (9)	C14—Ni2—C14^ii^	180.000 (1)
C15—C16—H16	126.0	C15—Ni2—C17	62.17 (6)
C17—C16—H16	126.0	C15^ii^—Ni2—C17	117.83 (6)
Ni2—C16—H16	112.9	C16—Ni2—C17	36.59 (6)
C22—C17—C18	120.18 (18)	C16^ii^—Ni2—C17	143.41 (6)
C22—C17—C16	132.02 (17)	C14—Ni2—C17	61.50 (5)
C18—C17—C16	107.78 (13)	C14^ii^—Ni2—C17	118.50 (5)
C22—C17—Ni2	132.89 (11)	C15—Ni2—C17^ii^	117.83 (6)
C18—C17—Ni2	74.18 (8)	C15^ii^—Ni2—C17^ii^	62.17 (6)
C16—C17—Ni2	57.84 (8)	C16—Ni2—C17^ii^	143.41 (6)
C19—C18—C17	119.91 (15)	C16^ii^—Ni2—C17^ii^	36.59 (6)
C19—C18—C14	132.77 (15)	C14—Ni2—C17^ii^	118.50 (5)
C17—C18—C14	107.31 (14)	C14^ii^—Ni2—C17^ii^	61.50 (5)
C19—C18—Ni2	134.13 (11)	C17—Ni2—C17^ii^	180.000 (1)
C17—C18—Ni2	71.90 (9)	C15—Ni2—C18	61.93 (6)
C14—C18—Ni2	59.08 (7)	C15^ii^—Ni2—C18	118.07 (6)
C20—C19—C18	118.66 (18)	C16—Ni2—C18	61.16 (6)
C20—C19—H19	120.7	C16^ii^—Ni2—C18	118.84 (6)
C18—C19—H19	120.7	C14—Ni2—C18	36.79 (5)
C21—C20—C19	121.4 (2)	C14^ii^—Ni2—C18	143.21 (5)
C21—C20—H20	119.3	C17—Ni2—C18	33.92 (5)
C19—C20—H20	119.3	C17^ii^—Ni2—C18	146.08 (6)
C22—C21—C20	121.16 (17)	C15—Ni2—C18^ii^	118.07 (6)
C22—C21—H21	119.4	C15^ii^—Ni2—C18^ii^	61.93 (6)
C20—C21—H21	119.4	C16—Ni2—C18^ii^	118.84 (6)
C21—C22—C17	118.71 (19)	C16^ii^—Ni2—C18^ii^	61.16 (6)
C21—C22—H22	120.6	C14—Ni2—C18^ii^	143.21 (5)
C17—C22—H22	120.6	C14^ii^—Ni2—C18^ii^	36.79 (5)
C14—C23—C25	112.06 (13)	C17—Ni2—C18^ii^	146.08 (5)
C14—C23—C26	110.38 (13)	C17^ii^—Ni2—C18^ii^	33.92 (5)
C25—C23—C26	108.07 (13)	C18—Ni2—C18^ii^	180.000 (1)

Symmetry codes: (i) −*x*+1, −*y*+2, −*z*+2; (ii) −*x*+2, −*y*+1, −*z*+1.

## References

[bb1] Allen, F. H. (2002). *Acta Cryst.* B**58**, 380–388. 10.1107/s0108768102003890 12037359

[bb6] Altomare, A., Cascarano, G., Giacovazzo, C., Guagliardi, A., Burla, M. C., Polidori, G. & Camalli, M. (1994). *J. Appl. Cryst.* **27**, 435.

[bb2] Bönnemann, H. (1985). *Angew. Chem. Int. Ed.* **24**, 248-262.

[bb3] Caddy, P., Green, M., Smart, L. E. & White, N. (1978). *J. Chem. Soc. Chem. Commun.* **19**, 839–841.

[bb4] Elschenbroich, Ch. (2008). *Organometallchemie*, 6th ed, p. 463. Wiesbaden: Teubner.

[bb5] Fontaine, F.-G. & Zargarian, D. (2004). *J. Am. Chem. Soc.* **126**, 8786–8794. 10.1021/ja048911m 15250732

[bb7] Gou, S., Hauptmann, R., Belaj, F. & Schneider, J. J. (2007). *Z. Kristallogr. New Cryst. Struct.* **222**, 363-634.

[bb8] Keller, E. (1999). *SCHAKAL99* University of Freiburg, Germany.

[bb9] Marder, T. B., Roe, D. C. & Milstein, D. (1988). *Organometallics*, **7**, 1451–1453.

[bb10] O’Connor, J. M. & Casey, C. P. (1987). *Chem. Rev.* **87**, 307–318.

[bb11] Oxford Diffraction (2010). *CrysAlis CCD* and *CrysAlis RED* Oxford Diffraction Ltd, Yarnton, England.

[bb12] Rerek, M. E. & Basolo, F. (1984). *J. Am. Chem. Soc.* **106**, 5908–5912.

[bb13] Rerek, M. E., Ji, L.-N. & Basolo, F. (1983). *J. Chem. Soc. Chem. Commun.* pp. 1208–1209.

[bb14] Sheldrick, G. M. (2008). *Acta Cryst.* A**64**, 112–122. 10.1107/S0108767307043930 18156677

[bb15] Turaki, N. N., Huggins, J. M. & Lebioda, L. (1988). *Inorg. Chem.* **27**, 424–427.

[bb16] Westrip, S. P. (2010). *J. Appl. Cryst.* **43**, 920–925.

[bb17] Xie, L.-Z., Sun, H.-M., Hu, D.-M., Liu, Z.-H., Shen, Q. & Zhang, Y. (2009). *Polyhedron*, **28**, 2585–2590.

